# Step-Wise Assembly, Maturation and Dynamic Behavior of the Human CENP-P/O/R/Q/U Kinetochore Sub-Complex

**DOI:** 10.1371/journal.pone.0044717

**Published:** 2012-09-18

**Authors:** Anja Eskat, Wen Deng, Antje Hofmeister, Sven Rudolphi, Stephan Emmerth, Daniela Hellwig, Tobias Ulbricht, Volker Döring, James M. Bancroft, Andrew D. McAinsh, M. Cristina Cardoso, Patrick Meraldi, Christian Hoischen, Heinrich Leonhardt, Stephan Diekmann

**Affiliations:** 1 Molecular Biology, FLI, Jena, Germany; 2 Department of Biology II, Center for Integrated Protein Science, Ludwig Maximilians University Munich, Planegg-Martinsried, Munich, Germany; 3 Institute of Biochemistry, ETH Zurich, Zurich, Switzerland; 4 Department of Biology,Technische Universität Darmstadt, Darmstadt, Germany; 5 Centre for Mechanochemical Cell Biology, Warwick Medical School, University of Warwick, Coventry, United Kingdom; University of Connecticut, Storrs, United States of America

## Abstract

Kinetochores are multi-protein megadalton assemblies that are required for attachment of microtubules to centromeres and, in turn, the segregation of chromosomes in mitosis. Kinetochore assembly is a cell cycle regulated multi-step process. The initial step occurs during interphase and involves loading of the 15-subunit constitutive centromere associated complex (CCAN), which contains a 5-subunit (CENP-P/O/R/Q/U) sub-complex. Here we show using a fluorescent three-hybrid (F3H) assay and fluorescence resonance energy transfer (FRET) in living mammalian cells that CENP-P/O/R/Q/U subunits exist in a tightly packed arrangement that involves multifold protein-protein interactions. This sub-complex is, however, not pre-assembled in the cytoplasm, but rather assembled on kinetochores through the step-wise recruitment of CENP-O/P heterodimers and the CENP-P, -O, -R, -Q and -U single protein units. SNAP-tag experiments and immuno-staining indicate that these loading events occur during S-phase in a manner similar to the nucleosome binding components of the CCAN, CENP-T/W/N. Furthermore, CENP-P/O/R/Q/U binding to the CCAN is largely mediated through interactions with the CENP-N binding protein CENP-L as well as CENP-K. Once assembled, CENP-P/O/R/Q/U exchanges slowly with the free nucleoplasmic pool indicating a low off-rate for individual CENP-P/O/R/Q/U subunits. Surprisingly, we then find that during late S-phase, following the kinetochore-binding step, both CENP-Q and -U but not -R undergo oligomerization. We propose that CENP-P/O/R/Q/U self-assembles on kinetochores with varying stoichiometry and undergoes a pre-mitotic maturation step that could be important for kinetochores switching into the correct conformation necessary for microtubule-attachment.

## Introduction

During mitosis, accurate chromosome segregation is essential for the correct transmission of the genetic material to the daughter cells. A multi-protein complex, the kinetochore, assembles at the centromere of each chromatid in order to mediate this function. Kinetochores contain an inner core that is present throughout the cell cycle [1], [2], and a set of outer kinetochore proteins that stably associate with the inner core during mitosis [3], [4]. The kinetochore is built from two major conserved protein networks, (1) the CCAN (constituitive centromere associated network) complex [5]–[13] which is associated to centromeric nucleosomes [5], [14]–[16] that consist of repetitive α-satellite DNA containing the histone H3 variant CENP-A [17], [18], and (2) the KMN network [7], [19]–[27] which directly connects the kinetochore to microtubules [3], [28], [29]. Functionally, the CCAN is required for the efficient recruitment of CENP-A into centromeric nucleosomes at the end of mitosis [6], [14], [30], [31] and the maintenance of centromeric chromatin, but is also involved in chromosome alignment, kinetochore fiber stability and bipolar spindle assembly [1], [2], [5], [6], [8], [32]–[34]. The CCAN was suggested to establish, in interphase, an inner kinetochore structure which functions as an assembly platform for KMN network proteins in mitosis, and only the KMN proteins then connect the inner kinetochore to microtubules [3]. However, ectopical CENP-T and -C alone are able to establish a functional outer kinetochore [16], [35] indicating that instead of being only a structural platform, the CCAN seems to be a regulator of the mitotic kinetochore-microtubule attachment [36].

The CCAN proteins CENP-U, -O, -P, -Q, and -R were identified as a CCAN subclass (named CENP-O class proteins) [3], [5], [6], [10], [37]. CENP-PORQU proteins are non-essential showing, when depleted, common mitotic defects and slower proliferation rates [6], [10], [33], [38]. Kinetochore localization of CENP-PORQU is interdependent [5], [10], [36]. In chicken DT40 cells, and when these genes are expressed in *E. coli*, CENP-O, -P, -U and -Q form a stable complex to which CENP-R can associate [10]. These data describe the CENP-PORQU complex as a stable unit which might function as a structural element in the CCAN. However, CENP-PORQU proteins have different protein specific functions: CENP-U [39] as well as CENP-Q [36] are able to bind to microtubules, only depletion of CENP-O seems to destabilise microtubule bundles at kinetochores influencing bipolar spindle assembly [34], [39], and CENP-U interacts with Hec1, an interaction negatively regulated by Aurora-B kinase [39]. In the complex, CENP-P is closely associated with CENP-O, and CENP-U binds to CENP-Q [10], [40]–[42]. In order to resolve these different views, we analysed protein binding, complex architecture and dynamics of the human kinetochore CENP-PORQU sub-complex by various *in vivo* techniques.

## Materials and Methods

### Plasmids

Plasmids pIC133, pIC190, pIC141, pIC140, and pIC235 encoding LAP-CENP-K, -Q, -P, -O, respectively -R fusion proteins were a kind gift of Dan Foltz and Iain Cheeseman. The full length cDNA clone of CENP-L, IRAUp969 EO882D, was from RZPD, Berlin, Germany). They were used for amplification of full length CENP-K, -L, -Q, -P, -O, and -R by PCR (Expand high fidelity^PLUS^ PCR System, Roche, Penzberg, Germany) applying forward primer 5′-GGGGACAAGTTTGTACAAAAAAGCAGGCTTCGAAAACCTGTATTTTCAGGGC*GCCACCATGG*GCATGAATCAGGAGGATTTAGATCC -3′ and reverse primer 5′- GGGGACCACTTTGTACAAGAAAGCTGGGTCTGATGGAAAGCTTCTAATCTTATT -3′ for CENP-K, forward primer 5′- GGGGACAAGTTTGTACAAAAAAGCAGGCTTCGAAAACCTGTATTTTCAGGGC*GCCACC*ATGGATTCTTACAGTGCACCAG -3′ and reverse primer 5′- GGGGACCACTTTGTACAAGAAAGCTGGGTCTCAATTTGAAAAATTGCCAGTTCTG for CENP-L, forward primer 5′- GGGGACAAGTTTGTACAAAAAAGCAGGCTTCGAAAACCTGTATTTTCAGGGCGCCACCATGGGCATGTCTGGTAAAGCAAATGCTTC -3′ and reverse primer 5′- GGGGACCACTTTGTACAAGAAAGCTGGGTAGATGCATCCAGTTTCTTATAGG -3′ for CENP-Q, forward primer 5′- GGGGACAAGTTTGTACAAAAAAGCAGGCTTCGAAAACCTGTATTTTCAGGGCGCCACCATGGACGCAGAGCTGGCAGA -3′ and reverse primer 5′- GGGGACCACTTTGTACAAGAAAGCTGGGTGTTGTTCTCCTCTGCACAAAGC -3′ for CENP-P, forward primer 5′- GGGGACAAGTTTGTACAAAAAAGCAGGCTTCGAAAACCTGTATTTTCAGGGCGCCACCATGGAGCAGGCGAACCCTTT -3′ and reverse primer 5′- GGGGACCACTTTGTACAAGAAAGCTGGGTGGAGACCAGACTCATATCCAAC -3′ for CENP-O, and forward primer 5′- GGGGACAAGTTTGTACAAAAAAGCAGGCTTCGAAAACCTGTATTTTCAGGGCGCCACCATGGGCATGCCTGTTAAAAGATCACTGAA -3′ and reverse primer 5- GGGGACCACTTTGTACAAGAAAGCTGGGTGTTTAAAATGGCTTTAAGGAATTCA -3′ for CENP-R. The CENP-Q, -P, -O, and -R harbouring linear PCR fragments were transferred into vector pDONR221 by BP recombination reaction (Invitrogen, Carlsbad, CA, USA). After verification by sequencing (MWG Biotech, Ebersberg, Munich, Germany), the genes were cloned by LR recombination reactions into various modified pFP-C and pFP-N (BD Biosciences, Clontech, Palo Alto, CA, USA) based Destination vectors. As the result we obtained expression vectors carrying the genes coding for CENP-Q, -P, -O, and -R fused to the C-termini as well as to the N-termini of EGFP and mCherry. In the constructed fluorescent proteins (FP)-CENP-Q, -P, -O, and -R, the amino acid (aa) linker between the fused proteins is SGTSLYKKAGFENLYFQGAT. Due to the cloning protocol, the aa sequence TQLSCTKW is added to the C-terminal ends of FP-CENP-Q, -P, -O, and -R. In the constructs where CENP-Q, -P, -O, and -R are fused to the N-termini of EGFP respectively mCherry, the (aa) linker is TQLSCTKWLDPPVAT. The cloning of CENP-U and -C [43] and CENP-N [44] have been described previously. Vector pIRES2 used for the simultaneous expression of EGFP and mRFP, was a friendly gift of J. Langowski (Heidelberg). For expression of a mRFP-EGFP fusion protein, we digested vector pmRFP-C1 with SnaBI and XmaI and ligated the resulting 1012 bps DNA fragment containing mRFP into a 7106 bp DNA obtained from a SnabI-XmaI digest of vector pH-G-C. In the resulting Gateway expression vector pH-mR-G-C, the amino acid linker between mRFP and EGFP is SGLRSRAQASNSAVDGTAGPVAT. Full length protein expression of the fusion constructs was confirmed by Western Blots.

### Live cell FRET measurements

FRET was measured by applying the acceptor photo-bleaching method using the FRET pair EGFP-mCherry. Co-transfected HEp-2 cells grown on coverslips were analyzed using a confocal laser scanning microscope (LSM 510 Meta) and a C-Apochromat 63×/1.2NA oil immersion objective (Carl Zeiss, Jena, Germany). EGFP fluorescence was excited with the Argon 488 nm laser line and analyzed using the Meta detector (ChS1+ChS2: 505–550 nm). mCherry fluorescence was excited with the 561 nm laser line (DPSS 561-10) and detected in one of the confocal channels using a 575–615 nm band-pass filter. To minimize cross talk between the channels, each image was collected separately in the multi-track-mode, i.e. both fluorophores were exited and recorded specifically and separately. Cells moderately expressing both fusion proteins with comparable expression levels were selected for analysis. Acceptor photo-bleaching was achieved by scanning a region of interest (ROI) including up to five centromeres of a nucleus 50 times (scans at 1.6 µsec pixel time) using the 561 nm laser line at 100% intensity. Bleaching times per pixel were identical for each experiment, however, total bleaching times varied depending on the size of the bleached ROIs. 4 donor and acceptor fluorescence images were taken before and up to 4 images after the acceptor photo-bleaching procedure to assess changes in donor and acceptor fluorescence. To minimize the effect of photo-bleaching of the donor during the imaging process, the image acquisition was performed at low laser intensities. To compare the time course of different experiments, donor intensities in the ROI were averaged and normalized to the intensity measured at the first time point after photo-bleaching, and acceptor intensities in the ROI were averaged and normalized to the mean intensity measured at time points 2–4 before photo-bleaching. The FRET efficiency was calculated by comparing the fluorescence intensity (I_DA_) before bleaching (in presence of the acceptor) with the intensity (I_D_) measured after bleaching (in the absence of the acceptor) according to E = 1−I_DA_/I_D_. The FRET efficiencies of numerous bleached and unbleached locations were compared by a paired t-test (α = 0.05). The difference between the means is a measure for the FRET-value, which was interpreted to have occurred when the paired t-test revealed a statistically significant difference between the two input groups with a p-value below 0.001. A p-value >0.001 was interpreted as an indication for insignificant FRET.

In two cases, the acceptor-bleaching FRET data were confirmed by additional fluorescence lifetime FLIM experiments. In FLIM experiments, the donor fluorescence lifetime was determined by time-correlated single photon counting (TCSPC) in living human HEp-2 cells. For donor fluorescence excitation, a pulsed picosecond diode laser (LDH Series, PicoQuant, Berlin, Germany) with a frequency of 20 MHz along with a dedicated driver (PDL Series, PicoQuant) was used. Via a fiber coupling unit, the excitation light was guided into a confocal laser scanning microscope (LSM 510 Meta). Laser power was adjusted to give average photon counting rates of 10^4^–10^5^ photons/sec (0.0001–0.001 photon counts per excitation event) or less to avoid pulse pile-up. Images of 256×256 pixels were acquired with a 63× C-Apochromat water immersion objective (NA 1.20, Carl Zeiss). Photons emitted by the sample were collected by the water immersion objective and detected by a single photon avalanche diode (PDM series, PicoQuant). The data were acquired by the PicoHarp 300 TCSPC module (PicoQuant) working in the TTTR mode (time-tagged time-resolved). To calculate the fluorescence lifetime, the SymPhoTime software package (v4.7, PicoQuant) was used. Selected areas of the images corresponding to single centromeres (resulting in the fluorescence lifetime histograms) or the sum of all centromeric regions were fitted by maximum likelihood estimation (MLE). Depending on the quality of a fit indicated by the value of χ^2^, a mono- or bi-exponential fitting model including background was applied. A model was rejected when χ^2^ exceeded a value of 1.5. In this way, the presence of scattered light in few measurements could be identified and separated. However, due to low photon numbers and too close time constants, the simultaneous presence of two different donor fluorescence lifetimes for complexes with donor-only and donor plus acceptor in one centromere could not be separated by a bi-exponential fit. A donor fluorescence lifetime obtained from a centromere in a cell co-expressing donor and acceptor molecules was considered to be significantly different from the control measurement, when the lifetime differed from the mean of the control values by >3 standard deviations. The FRET efficiency was calculated by comparing the donor fluorescence lifetime (τ_DA_) in the presence of the acceptor with the respective fluorescence lifetimes (τ_D_) of control measurements obtained in absence of an acceptor following E = 1−τ_DA_/τ_D_.

### F3H

BHK cells containing a *lac* operator repeat array [45] were cultured in DMEM medium with 10% FCS and seeded on coverslips in 6-well plates for microscopy. After attachment, cells were co-transfected with expression vectors for the indicated fluorescent fusion proteins and a LacI-GBP fusion [46], [47] using polyethylenimine (Sigma, St. Louis, USA). After about 16 hrs cells were fixed with 3.7% formaldehyde in PBS for 10 minutes, washed with PBST (PBS with 0.02% Tween), stained with DAPI and mounted in Vectashield medium (Vector Laboratories, Servison, Switzerland).

Samples were analyzed with a confocal fluorescence microscope (TCS SP5, Leica, Wetzlar, Germany) equipped with a 63×/1.4 numerical aperture Plan-Apochromat oil immersion objective as described [47]. DAPI, EGFP and mCherry were excited by 405 nm diode laser, 488 nm argon laser and 561 nm diode-pumped solid-state laser, respectively. Images were recorded with a frame size of 512×512 pixels.

### Cell culture, transfection and Western Blots

HeLa, HEp-2 and U2OS cells (ATCC, Manassas, USA) were cultured and Western blots were carried out as described [44], [48]. In order to determine cell cycle dependent CENP-O/Q/P levels, HEp-2 or HeLa cells were synchronised by double-thymidine block. Aliquots of equal cell numbers were taken after 2, 4, 6, 8 and 10 hrs after release and lysed. In the Western blot, CENP-O [33], CENP-P [36] and CENP-Q (Rockland, Gilbertsville, USA) are identified by specific primary antibodies which are then detected by fluorescently labelled secondary antibody (Molecular Probes, Eugene, USA). CENP-O/P/Q amounts are quantified by the ODYSSEY Infrared Imaging System (LiCor, Lincoln, USA) following the protocol of the manufacturer.

### Fluorescence Cross-Correlation Spectroscopy (FCCS)

FCCS analyses [49], [50] were performed at 37°C on an LSM 710 Confocor3 microscope (Carl Zeiss, Jena, Germany) using a C-Apochromat infinity-corrected 40×/1.2 NA water objective. U2OS cells were double transfected with vectors for the simultaneous expression of EGFP and mCherry fusion proteins and analysed. On cells expressing both fusion proteins at relatively low and comparable levels, we selected spots for the FCCS measurements in areas of the nucleoplasm which were free of kinetochores. For illumination of the EGFP-fusion proteins, we used the 488 nm laser line of a 25 mW Argon/2-laser (Carl Zeiss) and for simultaneous illumination of the mCherry fusion proteins a DPSS 561-10-laser (Carl Zeiss), both at moderate intensities between 0.2 and 0.5%. The detection pinhole was set to a relatively small diameter of 40 µm (corresponding to about 0.8 airy units). After passing a dichroic beam splitter for APDs (avalange photodiode detector; NTF 565), the emission of mCherry was recorded in channel 1 through a BP-IR 615–680 nm bandpath filter by an APD (Carl Zeiss), whereas the emission of EGFP was simultaneously recorded in channel 2 through a BP-IR 505–540 nm bandpath filter by a second APD. Before each measurement, we analysed possible crosstalk between the channels and used only cells without or with very little crosstalk. In addition, measurements with autocorrelation values below 1.06 for both, the mRFP channel as well as the EGFP channel, were not further analysed. For the measurements, 10×10 time series of 10 sec each were simultaneously recorded for mCherry and for EGFP. After averaging, the data were superimposed for fitting with the Fit-3Dfree-1C-1Tnw model of the ZEN-software (Carl Zeiss), a diffusion model in three dimensions with triplet function. Applying this procedure, we obtained autocorrelations of channels 1 and 2 as well as the cross-correlation of channels 1 versus channel 2. Before starting a set of experiments, the pinhole was adjusted. As negative control, U2OS cells were transfected with vector pIRES2, separately expressing EGFP and mRFP as single molecules with fluorescence intensities comparable to those in the FCCS analysis with CENP fusion proteins. As a positive control, U2OS cells were transfected with pH-mR-G-C expressing a mRFP-EGFP fusion protein, again with fluorescence intensities comparable to those in the FCCS analysis with CENP fusion proteins.

### Cellular imaging

*In vivo* and *in situ* cellular imaging including immuno-fluorescence, SNAP-tag analysis, FRAP, RICS and cell cycle sychronisation were conducted as described in Orthaus et al. [48], [51], Hellwig et al. [43], [44] and McClelland et al [8]. For immuno-flourescence, primary antibodies were used at 1∶250 (PCNA), 1∶300 (anti-CENP-Q), 1∶250 (CREST) with DAPI at 1∶2000.

## Results

### CENP-O class proteins form a tightly packed complex

In chicken DT40 cells, the CENP-O class proteins form a tight kinetochore sub-complex [10]. Here we analysed the CENP-O class protein packaging at kinetochores in living human cells by measuring which proteins are in close proximity. We tagged all five CENP-O class proteins with fluorescent proteins, either EGFP or mCherry, at either termini, and confirmed by live cell imaging in human U2OS cells that all tagged CENP-O class proteins localise to kinetochores during interphase and mitosis, consistent with published results [5], [6], [10], [33], [36], [39]. This kinetochore localisation was independent of which terminus of the CENP proteins was tagged.

**Figure 1:**
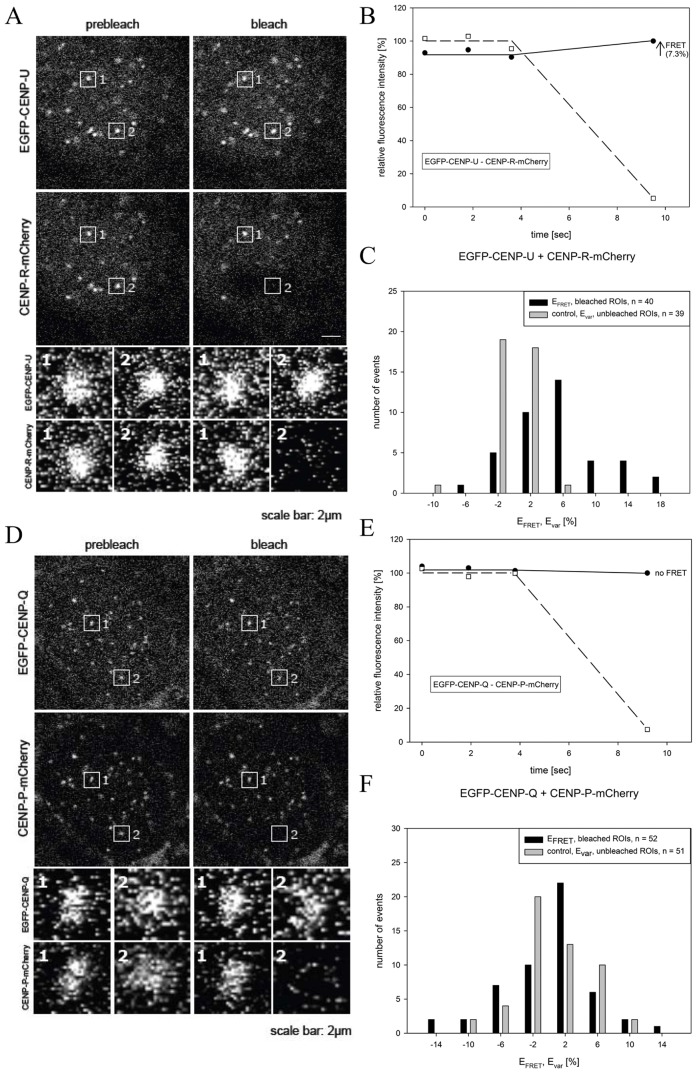
Acceptor-bleaching FRET of the protein pairs EGFP-CENP-U/CENP-R-mCherry (FRET signal) and EGFP-CENP-Q/CENP-P-mCherry (no FRET signal). Typical HEp-2 cell nuclei are displayed in (A) and (D) showing centromere location of all four CEN proteins. Two of these locations, spot 1 and spot 2 in each of the two graphs, were selected for fluorescence intensity analysis before and after acceptor bleaching (see enlargements below). Spot 1 served as control and showed no detectable intensity change. At spot 2, the acceptor fluorophore mCherry was bleached (compare pre-bleach and post-bleach in (A) and (D)). In (B) and (E), the time course of the fluorescence intensity of the donor and the acceptor of both FRET pairs are shown. The acceptor intensities in the ROI (“region of interest”; open squares) were averaged and normalized to the mean intensity measured at the three time points before bleaching. The donor intensities in the ROI were averaged and normalized to the intensity measured at the time point after bleaching. Bleaching of the acceptor resulted in a fluorescence intensity increase of the donor (black dots) for EGFP-CENP-U (B) indicating the presence of FRET (see arrow), but no fluorescence intensity increase for EGFP-CENP-Q (E) indicating the absence of FRET. (C) and (F): Donor fluorescence intensity variation observed during acceptor bleaching normalized to the intensity measured at the first time point after bleaching. Control: spot 1 (acceptor not bleached) yielding E_var_ (grey bars), FRET measurement: spot 2 (acceptor bleached) yielding E_FRET_ (black bars). For protein pairs indicated, number of observed single cases (grouped into E_var_ or E_FRET_ value ranges of 4%) displayed versus values of E_var_ or E_FRET_. (C) EGFP-CENP-U (donor) and CENP-R-mCherry (acceptor): distribution of E_FRET_ (40 bleached kinetochores) is clearly distinct from the distribution of E_var_ (39 non-bleached kinetochores) indicating FRET. (F): EGFP-CENP-Q (donor) and CENP-P-mCherry (acceptor): distribution of E_FRET_ (52 bleached kinetochores) superimposes the distribution of E_var_ (51 non-bleached kinetochores) indicating no FRET.

Then, by FRET we measured the proximity between chromophores tagged to CENP-O class proteins. FRET between the donor fluorophore (here: EGFP) and the acceptor fluorophore (here: mCherry) can only generate a positive result when the distance between donor and acceptor is less than ∼10 nm. When FRET occurs, both the intensity and lifetime of the donor fluorescence decrease while the intensity of the acceptor emission increases. We measured the FRET donor fluorescence intensity with or without photo-inactivation of the acceptor (acceptor-photo-bleaching FRET, AB-FRET) and, in order to confirm our AB-FRET results, in two cases also the donor fluorescence lifetime (FLIM). In AB-FRET, the acceptor chromophore is destroyed by photo-bleaching, thereby preventing FRET from the donor to the acceptor. Thus, when the donor is in close proximity to the acceptor (sufficient for FRET, <10 nm), photo-bleaching of the acceptor results in an observable increase in donor fluorescence. In our experiments, two separate kinetochore locations were identified in each image (marked “1” and “2”; Fig. 1A and 1D). In spot “2” the acceptor (CENP-R-mCherry (Fig. 1A), CENP-P-mCherry (Fig. 1D)) was photo-bleached, while spot “1” was not photo-bleached, serving as an internal control for any non-FRET effects. During bleaching of the acceptor (CENP-R-mCherry) in spot 2, the donor (EGFP-CENP-U) fluorescence intensity significantly increased indicating that FRET occurred between EGFP-CENP-U and CENP-R-mCherry (Fig. 1B). Careful quantification indicated that such FRET transfer occurred in 60% of the cases, yielding a FRET efficiency E_FRET_ between 6 and 18% (40 bleached spots in 18 cells, black bars in Fig. 1C). The unbleached control spots show a narrow fluorescence variation E_var_ around zero (39 bleached spots in 18 cells, grey bars in Fig. 1C). The E_FRET_ distribution is significantly different from the E_var_ control distribution (p<0.001). Such experiments demonstrated that the majority of pairs gave a positive FRET signal suggesting that the CENP-PORQU subunits are closely associated (see Table 1). Importantly, a number of pairs did not show FRET: We detected no FRET between EGFP-CENP-Q/CENP-P-mCherry (Fig. 1D–F). Here, after acceptor-bleaching, the donor fluorescence did not increase (Fig. 1E) and the distribution of the E_FRET_ values (black bars, Fig. 1F) superimposes with the distribution of the E_var_ control values (grey bars). Furthermore, we did not observe FRET between EGFP-CENP-P/CENP-O-mCherry and between EGFP-CENP-U/CENP-O-mCherry (see Table 1). For CENP-Q-EGFP and mCherry-CENP-P and for CENP-P-EGFP and mCherry-CENP-O, we confirmed these results by measuring FRET at kinetochores in the lifetime domain (FLIM) by time-correlated single photon counting (TCSPC) using the same fluorescent protein FRET pair EGFP-mCherry. This approach is less error prone compared to acceptor-bleaching FRET in the intensity domain, however, it is considerably more elaborate and time-consuming. We determined the CENP-Q-EGFP donor lifetime in the absence of an acceptor as τ = 2.45±0.10 nsec. When the acceptor is close, the donor life time decreases due to energy transfer to the acceptor: for CENP-Q-EGFP/mCherry-CENP-P we measured τ = 2.08±0.04 nsec and for CENP-P-EGFP/mCherry-CENP-O we measured τ = 2.16±0.05 nsec. The FLIM results (marked by “F” in Table 1) indicate the proximity between CENP-Q and -P as well as between CENP-P and -O and confirm our acceptor-bleaching FRET data. We conclude that in human cells at kinetochores, CENP-O class proteins are in close proximity to one another. In earlier studies we had detected FRET between the CENP-U N-terminal region and the N-termini of CENP-B and CENP-I, but not to the N-termini of CENP-A and CENP-C [43].

**Table 1:** FRET interactions between CENP-O class proteins.

EGFP fusion	mCherry fusion	p	FRET
EGFP-CENP-P	mCherry-CENP-O	<0.001	++
EGFP-CENP-P	CENP-O-mCherry	0.093	−
CENP-P-EGFP	mCherry-CENP-O	<0.001	++F
CENP-P-EGFP	CENP-O-mCherry	<0.001	++
EGFP-CENP-Q	mCherry-CENP-O	<0.001	++
EGFP-CENP-Q	CENP-O-mCherry	<0.001	++
EGFP-CENP-Q	mCherry-CENP-P	<0.001	++
EGFP-CENP-Q	CENP-P-mCherry	0.724	−
EGFP-CENP-Q	mCherry-CENP-Q	<0.001	++
CENP-Q-EGFP	CENP-Q-mCherry	<0.001	++
CENP-Q-EGFP	mCherry-CENP-P	<0.001	++F
EGFP-CENP-U	mCherry-CENP-O	<0.001	++
EGFP-CENP-U	CENP-O-mCherry	0.655	−
EGFP-CENP-U	mCherry-CENP-P	0.003	+
EGFP-CENP-U	CENP-P-mCherry	<0.001	++
EGFP-CENP-U	mCherry-CENP-Q	<0.001	++
EGFP-CENP-U	mCherry-CENP-R	<0.001	++
EGFP-CENP-U	CENP-R-mCherry	<0.001	++
CENP-U-EGFP	mCherry-CENP-P	<0.001	++
EGFP-CENP-B	CENP-Q-mCherry	<0.001	++
EGFP-CENP-O	mCherry-CENP-K	<0.001	++
CENP-O-EGFP	mCherry-CENP-K	0.004	+
EGFP-CENP-R	mCherry-CENP-K	<0.001	++
CENP-R-EGFP	mCherry-CENP-K	<0.001	++
EGFP-CENP-U	mCherry-CENP-K	<0.001	++
CENP-U-EGFP	mCherry-CENP-K	0.167	−
EGFP-CENP-U	mCherry-CENP-U	<0.001	++
CENP-N-EGFP	mCherry-CENP-K	<0.001	++

The FRET pair EGFP-mCherry is used. “F” indicates that for these fusions FRET was detected also by FLIM. ++: strong FRET, +: weak FRET, −: no FRET.

If the orientation of the fluorophore dipole moment of the acceptor relative to that of the donor were known, or at least one of them would rotate freely faster than nanoseconds, a more detailed distance between donor and acceptor could be deduced from the measured E_FRET_ values. In our live cell experiments however, this information is not available to us. We therefore do not deduce defined distance values but interpret the appearance of FRET as an indication that donor and acceptor chromophores are close to one another within 10 nm. Our FRET data depend on which protein terminus is tagged: if the two protein termini are clearly separated in space, a fluorophore fused to one terminus might show FRET to another protein while the fluorophore fused to the other terminus might not. In a number of cases, we could not detect FRET between two fusion proteins. Measuring no FRET signal might either be due to donor and acceptor fluorophores being distal (>10 nm) or, alternatively, that donor and acceptor dipole moments are oriented relative to one another in an unfavorable way so that FRET cannot occur although donor and acceptor are close. Therefore, observing no FRET signal cannot be used for structural information.

### PORQU undergoes a post-loading oligomerisation step

**Figure 2:**
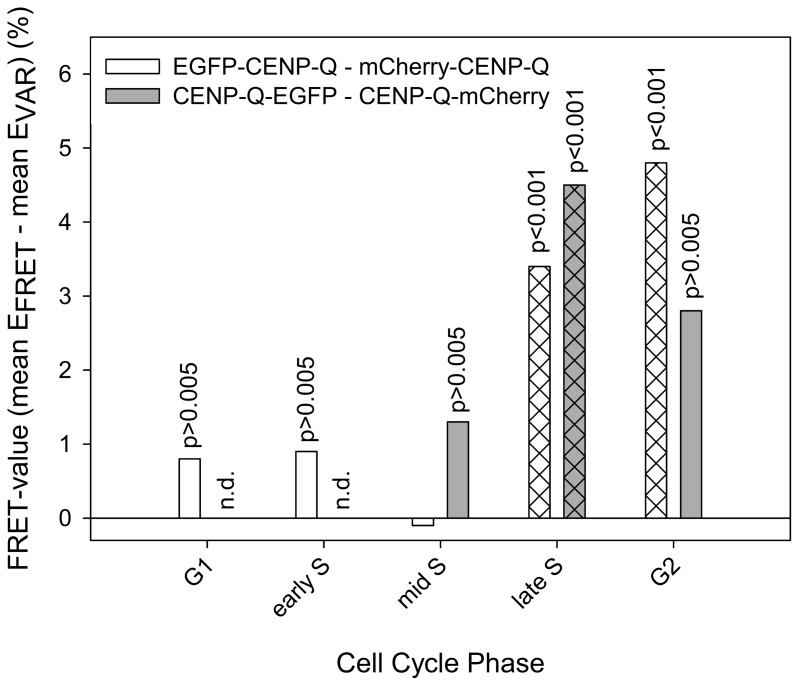
Cell cycle-dependent FRET between CENP-Q C- (grey bars) and N-termini (white bars). In late S-phase and G2, significant FRET is observed (p<0.001). In G1, early and mid S-phase, no FRET is observed (p>0.005).

Recombinant CENP-Q that is expressed and purified form *E. coli* lysates, exists as a soluble homo-octameric complex [36]. We therefore asked if CENP-Q oligomerises at kinetochores in living human cells. Indeed, we observed FRET at kinetochores between the N-termini of CENP-Q and between its C-termini in interphase cells, suggesting that CENP-Q oligomerises when kinetochore-bound. In order to find out when in the cell cycle CENP-Q oligomerizes, we carried out cell cycle dependent FRET measurements between C- and N-termini of CENP-Q (see Table 1, Fig. 2). U2OS cells were synchronised by double thymidine block and released into S-phase. Subsequent cell cycle phases were identified by CENP-F and PCNA staining. We found no FRET in G1, early and mid S-phase, however, we detected a significant FRET signal in late S-phase for both, the CENP-Q N- and C-termini, and in G2 for the CENP-Q N-termini (Fig. 2). Consistent with this, quantitative immuno-flourescence demonstrates that CENP-Q protein levels increase at kinetochores during S-phase and become maximal in late S-phase (see below). We also detected a FRET proximity between two CENP-U N-termini at kinetochores in late but not in early or middle S-phase (data not shown).

### PORQU proteins show multiple pair-wise interactions

**Figure 3:**
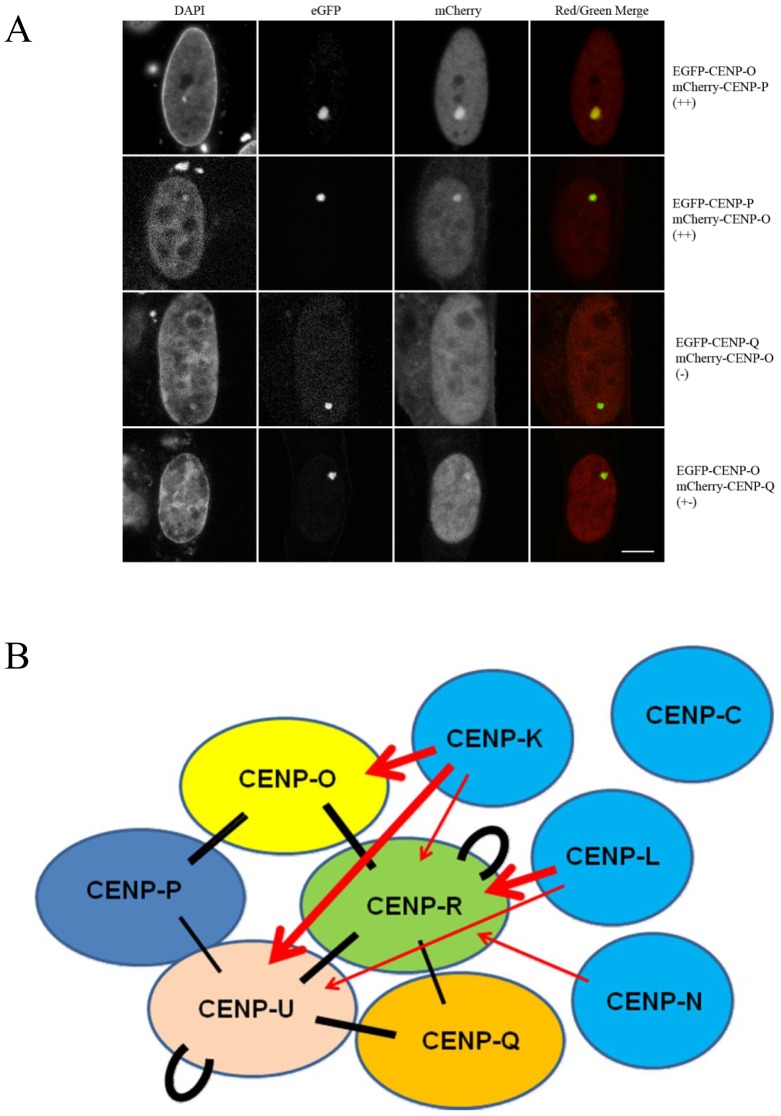
Centromere protein interactions analyzed by F3H assay. (A) EGFP tagged centromere proteins (bait, green) were recruited to the *lac* operator repeat array by the GFP-binding protein fused to the Lac repressor (LacI-GBP). Co-expressed mCherry tagged centromere proteins (prey, red) may either interact with the GFP-tagged protein (yellow in the overlay) or may not interact resulting in a disperse distribution. Upper two rows: interaction between EGFP-CENP-O and mCherry-CENP-P, EGFP-CENP-P and mCherry-CENP-O. Lower two rows: EGFP-CENP-Q did not interact with and recruit mCherry-CENP-O to the *lac* spot, but shows a weak interaction in the reverse combination. For all results see Table 1. Bar: 5 µm. (B): Strong F3H interactions are displayed (++: thick lines, +: thin lines). Black bars: interactions between CENP-PORQU proteins, red arrows: recruitment of CENP-PORQU proteins by CENP-K, -L, and -N. CENP-C is not able to recruit any of the CENP-PORQU proteins (data see Table 2).

Then we asked which of the CENP-O class proteins is able to interact with other protein members of this class. In the mammalian three-hybrid (F3H) assay applied here [46], [47], EGFP tagged CENP-O class proteins (bait) were recruited to the *lac* operator repeat array by the GFP-binding protein fused to the Lac repressor (GBP-LacI) forming a green spot in the nucleus (Fig. 3). Co-expressed mCherry-tagged CENP-O class proteins (prey) may either interact with the EGFP-tagged protein at the *lac* operator array (visible as red spot and yellow in the overlay) or may not interact resulting in a disperse distribution. For each mCherry fusion, EGFP was used to control for unspecific interactions. In the upper two rows of Fig. 3A, the clear interaction between EGFP-CENP-O and mCherry-CENP-P as well as EGFP-CENP-P and mCherry-CENP-O are shown. The lower two rows show the corresponding results for CENP-O and CENP-Q. While EGFP-CENP-Q did not interact with and recruit mCherry-CENP-O to the *lac* spot, we found a very weak interaction in the reverse combination. Such differences for reverse combinations might be explained by sterical hindrance at the interaction site due to the attachment to GBP-LacI for one but not the other tagged terminal region. All results of this F3H interaction assay are listed in Table 2. The results of this CENP-O class protein interaction analysis indicate strong interactions between particular members of this class (Fig. 3B). CENP-O, -P and -Q each are able to strongly recruit and thus specifically bind two, while CENP-U and -R are able to recruit three of the remaining four proteins. In addition, CENP-U and CENP-R are able to bind to themselves. We detected homo-interaction of CENP-R also by a Yeast-two-Hybrid (Y2H) assay. CENP-U binding to itself is supported by our FRET data indicating close proximity between CENP-U N-termini in late S-phase (see above). Our data that CENP-P is closely associated with CENP-O, and CENP-U with CENP-Q, agree well with published results [10], [41], [42]; here we detected an additional weaker interaction between CENP-Q and CENP-R. However, none of the CENP-O class proteins is able to recruit all four other proteins of this class which would be expected when the complex pre-forms in the nucleoplasm. Furthermore, ectopic recruitment to the *lac* operator repeat array obviously is not strong enough to enable indirect binding: e.g. CENP-L recruits CENP-R, and CENP-R recruits CENP-Q, but CENP-L is not able to attrack CENP-Q to this site. Thus, this F3H assay is, to a large extent, specific for direct interactions.

**Table 2:** F3H analysis of CENP-O class protein interactions.

mCherry\EGFP	CENP-O	CENP-P	CENP-Q	CENP-R	CENP-U	CENP-K	CENP-L	CENP-N	CENP-C
CENP-O	−	++	−	++	−	+	−	−	−
CENP-P	+	−	+−	+−	+	−	−	−	−
CENP-Q	+−	−	−	+	+	−	−	−	−
CENP-R	++	+−	+	++	++	+−	++	−	−
CENP-U	−	+	++	++	++	++	+−	−	−
CENP-K	++	−	+−	+	++	+−	−	−	−
CENP-L	−	−	+−	+	+	+−	−	−	+−
CENP-N	−	−	−	+	+−	−	−	−	−
CENP-C	−	−	−	−	−	−	−	−	−

GFP-tagged CENP-O class proteins, CENP-K, -L, -N and -C (rows) were bound to ectopic chromosomes sites. When RFP-tagged CENP-O class proteins, CENP-K, -L, -N and -C (lines) were recruited to these proteins, this was visible by a yellow dot. Signal intensity at the nuclear spot was used an indicator for interaction strength. ++, +: strong interaction; +−: weak interaction; −: no interaction.

### CENP-PORQU subcomplex contacts other CCAN proteins

Kinetochore localization is determined by CENP-A which is recognized by CENP-N and CENP-C [14]–[16]. CENP-L binds to the C-terminal region of CENP-N *in vitro* [14] and CENP-K kinetochore localisation depends on the presence of CENP-N and -C [5], [8], [14], [52]. We therefore asked, if these kinetochore proteins, being directly or closely linked to CENP-A, are able to recruit single CENP-PORQU proteins or the whole complex to an ectopic chromatin site in human cell nuclei *in vivo*. We studied the interaction of CENP-C, -L, -K and -N with CENP-PORQU proteins by F3H; the results are listed in Table 2 and displayed in Fig. 3B. CENP-N shows binding to CENP-R and some weak binding to CENP-U, however, only for mCherry-tagged CENP-N (prey) while EGFP-tagged CENP-N (bait) does not show any interaction with CENP-PORQU proteins. CENP-L shows strong binding to CENP-R and moderate binding to CENP-U (strong in one, weak in the other version; see Table 2) and very weak binding to CENP-C (only in one orientation). Furthermore, RFP-tagged CENP-L also shows weak interactions with CENP-Q and CENP-K. Next to CENP-L, also CENP-K shows strong interactions with CENP-PORQU proteins: CENP-K strongly interacts with CENP-O and -U, moderately with CENP-R (strong in one, weak in the other version; see Table 2), and in one version weakly with CENP-Q. CENP-K also weakly binds to itself. By Y2H we detected an interaction between CENP-K and CENP-O, consistent with results of McClelland et al. [8], and an interaction between CENP-K and CENP-H, supporting data of Qui et al. [53], however no interaction had been detected by Y2H between CENP-O and either CENP-H or CENP-N [8]. We thus conclude that to some extend CENP-N, but more efficiently CENP-L and even more so CENP-K mainly recruit CENP-O, -U and -R to kinetochores but much less so CENP-Q, and not CENP-P. This finding agrees with results of Okada et al. [6] who observed in human and DT40 cells that the localization of CENP-O, -P, -Q and -H was disrupted in CENP-K and CENP-L depleted cells. Our results extend their observations by identifying the pairwise interactions responsible for the observed data: Potentially CENP-P and CENP-Q are disrupted from CENP-K and -L depleted cells due to being members of the CENP-PORQU complex and not due to specific protein-protein interactions. Similarly, the dependence of CENP-U kinetochore localization on the presence of CENP-H and -I [38] might be explained by CENP-H and -I being required for CENP-K binding which then recruits the CENP-PORQU complex. F3H yields more direct data on protein-protein interactions than depletion experiments which by their very nature also influence the presence of proteins down-stream of the depleted protein.

We observed no recruitment to the ectopic chromatin site of any CENP-PORQU protein by CENP-C. Furthermore, CENP-L and -N do not recruit all five CENP-PORQU proteins, again indicating that the CENP-PORQU complex does not pre-form in the nucleoplasm.

We confirmed these F3H results by FRET studies. We measured the close neighbourhood of CENP-K to several CENP-PORQU proteins and to CENP-N, and found proximities between the N-terminus of CENP-K with both termini of CENP-R, the N-termini of CENP-O and -U and to the C-terminus of CENP-N (see Table 1). These results place CENP-K inbetween CENP-N and the CENP-PORQU proteins.

### PORQU does not preassemble in the cytoplasm

**Figure 4:**
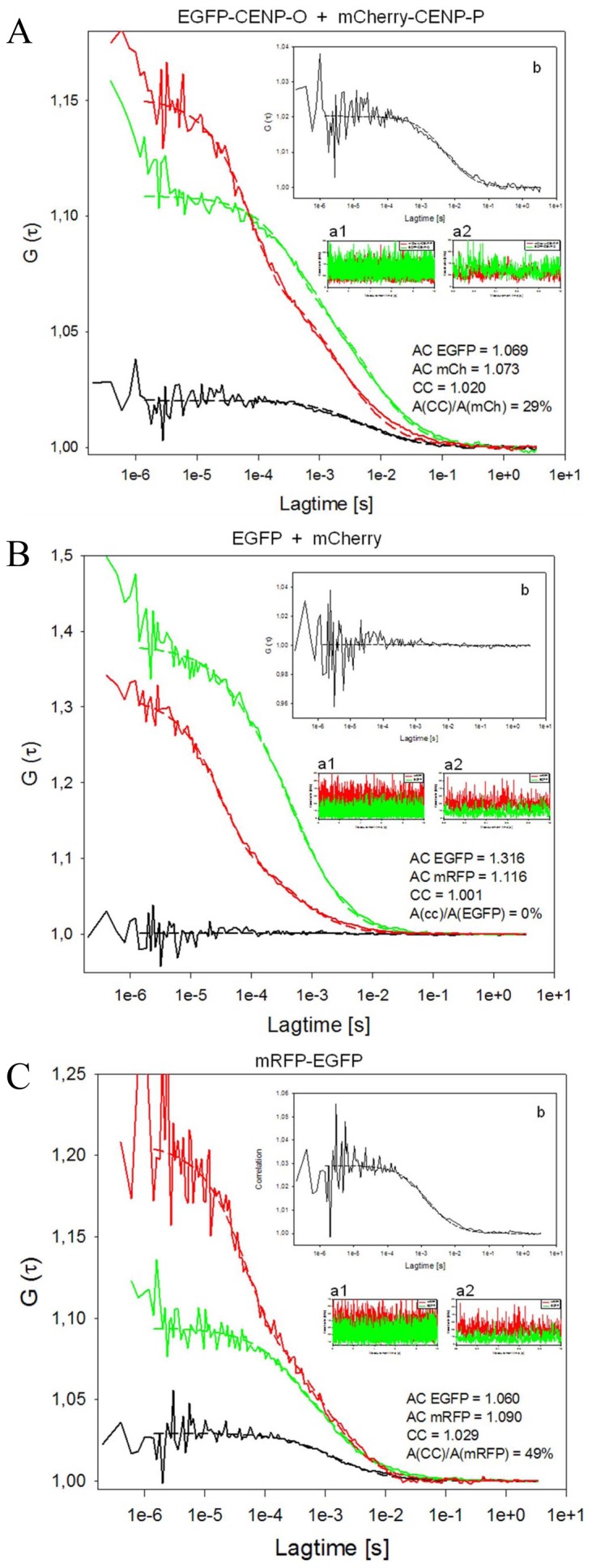
FCCS measurements displaying G versus lag time. Red: mCherry (A, B) or mRFP (C), green: EGFP, black: auto-correlation. Count rates are displayed over 10 sec (inserts a1) indicating the absence of photobleaching, and 1 sec (inserts a2) indicating the absence of larger protein aggregates. The cross-correlation analyses are amplified in inserts b. (A) EGFP-CENP-O and mCherry-CENP-P indicate complex formation in the nucleoplasm (amplitude of cross-correlation/amplitude of mCherry signal: 29%). The amplitude of the cross-correlation curve A(CC), relative to the diffusion-related amplitude of one of the autocorrelation curves A(AC) of EGFP or mCherry, is a measure of binding or dynamic colocalization [49], [50]. According to this ratio of amplitudes A(CC)/A(AC), up to 20–30% of nucleoplasmic CENP-O and -P are hetero-dimers. Count rates were recorded simultaneously for both fluorophores. The count rate detected in a 10 sec measurement (insert a1) demonstrates the absence of photobleaching, while the count rate in a 1 sec resolution time scale (insert a2) indicates the absence of larger protein aggregates. The autocorrelations yielded 1.069 and 1.073 for EGFP-CENP-O and mCherry-CENP-P, respectively. The cross-correlation analysis (with a magnified scale of G(τ); insert b) resulted in a correlation of 1.02 indicating that 29% of the molecules are co-migrating in the nucleoplasm. (B) EGFP and mCherry expressed as single non-fused proteins (negative control) do not show any cross-correlation (A(CC)/A(EGFP) = 0%). The count rates (inserts a1 and a2) indicate the absence of photobleaching and larger proteins. The autocorrelations yielded 1.316 and 1.116 for EGFP and mRFP, respectively. The cross-correlation curve (with a magnified scale of G(τ), insert b) resulted in a value of 1.001 indicating the absence of any complexation between EGFP and mRFP. (C) mRFP-EGFP fusion protein (positive control) shows cross-correlation (A(CC)/A(mRFP) = 49%). The count rates indicate that photobleaching and the presence of larger protein aggregates can be excluded (inserts a1, a2) and that the autocorrelations of EGFP (1.06) and mRFP (1.09) were comparable to the values obtained for EGFP-CENP-O and mCherry-CENP-P. Cross-correlating the two channels against each other, we obtained a value of 1.029 indicating that about 50% of the molecules are detected as a complex (with a magnified scale of G(τ) in insert b).

In order to analyse CENP-PORQU complex pre-assembly, in interphase we measured the mobility of the five CENP-O class proteins in the nucleoplasm of human U2OS cells by Raster Image Correlation Spectroscopy (RICS) [54] and found fast mobility between 4.7 and 5.9 (±15%) µm^2^/sec. The proteins are thus more mobile than other inner kinetochore proteins [44], [55]. The experimental variation of the measured mobilities, however, does not allow for a conclusion on multimerisation. We therefore performed Fluorescence Cross-Correlation Spectrometry (FCCS) studies to determine if CENP-O class proteins form hetero-dimers in the nucleoplasm. In double-transfected U2OS cells we analysed various protein pairs: EGFP-CENP-O/mCherry-CENP-P, EGFP-CENP-P/mCherry-CENP-Q, EGFP-CENP-R/mCherry-CENP-Q, EGFP-CENP-Q/mCherry-CENP-Q, CENP-O-EGFP/mCherry-CENP-Q, EGFP-CENP-U/mCherry-CENP-Q, EGFP-CENP-R/mCherry-CENP-R, EGFP-CENP-R/CENP-R-mCherry, CENP-U-EGFP/CENP-U-mCherry, CENP-U-EGFP/mCherry-CENP-U and EGFP-CENP-U/mCherry-CENP-O. For these protein pairs we found unequivocal cross-correlation only between CENP-O and CENP-P. From 12 cells, all 12 showed cross-correlation indicating that CENP-O and CENP-P move together, i.e. they are part of one and the same complex in the nucleoplasm outside kinetochores. The cross-correlation analysis (Fig. 4A) resulted in a correlation of 1.020 (Fig. 4A, insert b) indicating that 29% of the molecules are co-migrating in the nucleoplasm. As negative control, U2OS cells were analysed separately expressing EGFP and mRFP as single molecules. The cross-correlation curve (Fig. 4B) resulted in a value of 1.001 (Fig. 4B, insert b) indicating the absence of any complexation between EGFP and mRFP. As a positive control, U2OS cells were transfected with pH-mR-G-C expressing a mRFP-EGFP fusion protein. Cross-correlating the two channels against each other, we obtained a value of 1.029 indicating that about 50% of the molecules are detected as a complex (Fig. 4C). We obtained similar cross-correlation values for the fusion EGFP-mCherry, in agreement with results of Kohl et al. [56]. For such fusion proteins, 100% cross correlation should be observed. The lower value of 50% could be explained by a much slower maturation and lower stability of mRFP compared to EGFP: EGFP molecules bound to an immature mRFP are interpreted by FCCS as free molecules. Thus, cross-correlation values seem to underestimate the percentage of co-migrating molecules. Consequently, hetero-dimerisation of EGFP-CENP-O and mCherry-CENP-P probably is higher than the calculated 20–30%, we estimate 40–60%.

In 2 out of 12 analyzed cells, a weak cross-correlation (∼10%) was observed for CENP-Q and CENP-R indicating that in a few cases CENP-Q and CENP-R co-migrate in the nucleoplasm outside kinetochores. The other analyzed protein pairs showed no cross-correlation demonstrating that the CENP-PORQU complex does not pre-form in the nucleoplasm outside kinetochores. CENP-R and CENP-U are able to bind to themselves at an ectopic chromatin site (see above). However, by FCCS we did not detect any cross correlation, clearly indicating that these proteins do not stably aggregate in the nucleoplasm. Recombinant CENP-Q can oligomerise to octamers [36] and, when kinetochore-bound, oligomerises in late S-phase, as detected by FRET (see above). In the nucleoplasm, however, CENP-Q does not form di- or multimers, as shown here by FCCS. This FCCS result is confirmed by the absence of a FRET signal between two tagged CENP-Q in the nucleoplasm outside kinetochores (data not shown).

These data show that, with the two exceptions CENP-O/-P and CENP-Q/-R, the pairwise CENP-O class protein interactions detected by F3H do not result in a homo- or hetero-dimerisation of these proteins stable enough for FCCS detection. Since these proteins do not pre-aggregate, they must enter the nucleoplasm as single proteins. CENP-O, -P, -Q and -R are small enough (molecular weights <34 kDa) for not needing a nuclear localisation domain (NLS) for entering the nucleus. Only CENP-U is larger (47.5 kDa) and indeed contains two NLS [57], [58].

### PORQU loads onto kinetochores in S-phase and form a stable subcomplex

**Figure 5:**
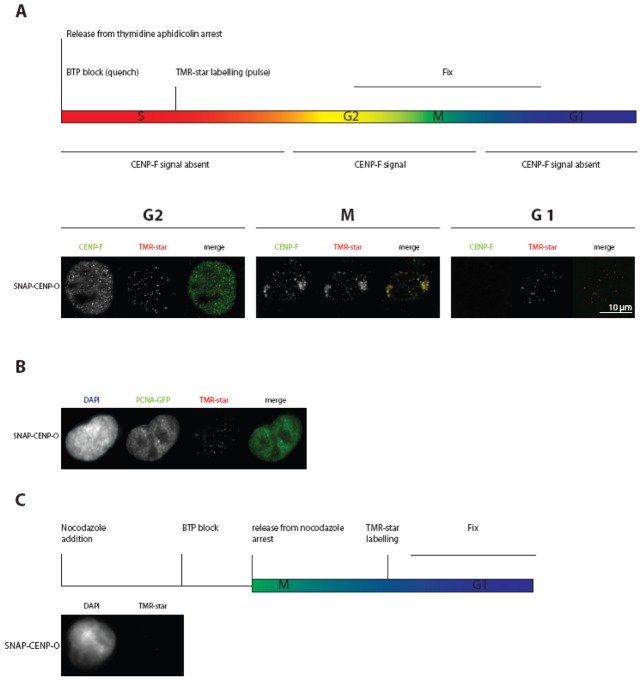
CENP-O loading to kinetochores measured by the SNAP-tag technology. (A) Top: schematic representation of the performed experiment. Below: representative images of cells showing TMR-star fluorescence for SNAP-CENP-O in G2, M-phase and the following G1. Cell cycle phases G2 (CENP-F staining of the whole nucleus) and mitosis (specific kinetochore binding of CENP-F) are clearly identified. (B) The same experiment as in (A) was performed with U2OS cells stably expressing PCNA-GFP. SNAP-CENP-O fluorescence appears at kinetochores in late S-phase as judged from cellular PCNA distributions. (C) Top: schematic representation of the performed experiment. Below: representative images of cells expressing SNAP-CENP-O showing no fluorescence at kinetochores during G1. CENP-O is thus loaded to kinetochores in S-phase.

We next asked when during the cell cycle the CENP-PORQU complex assembles. By applying SNAP-tag technology, we determined at which cell cycle phase CENP-O is loaded to the kinetochore. The SNAP protein tag can catalyze the formation of a covalent bond to a benzyl-guanine moiety coupled to different fluorescent or non-fluorescent membrane-permeable reagents [59]. This tag allows pulse-chase experiments at a single protein level. Consistent with previous data [60], we detected TMR-star fluorescence on SNAP-CENP-A only in G1 cells, confirming that CENP-A is specifically loaded in G1, while we observed a time window of mid G1 to G2 for CENP-N binding and loading to kinetochores [44]. Here we transfected a SNAP-CENP-O construct. After double-thymidine and aphidicolin block release and applying the same protocol, we found SNAP-CENP-O present at kinetochores of G2 cells (Fig. 5A), indicating that CENP-O is loaded onto kinetochores in or before G2. To extend our temporal analysis to further phases of the cell cycle, we repeated these experiments in U2OS cells since these cells have a longer cell cycle: 12 hrs after release and following the same experimental procedure, U2OS cells can be analysed in late-S-phase. Here, TMR-star fluorescence for SNAP-CENP-O was already detected in late S-phase as judged by PCNA-GFP fluorescence (Fig. 5B). Thus, CENP-O assembles at kinetochores already in late S-phase or earlier. Finally, to measure the earliest time point at which CENP-O can assemble into kinetochores, SNAP-CENP-O transfected HeLa cells were arrested in mitosis for 12 hrs by a nocodazole block and quenched with BTP for 30 min. 4 hrs after quenching, the cells were released from nocodazole arrest. Further 5 hrs later, SNAP-tagged CENP-O was fluorescently labelled with TMR-star for 30 min and fixed for examination. No TMR-star fluorescence was detected indicating that SNAP-CENP-O is not loaded in G1 (Fig. 5C). Overall these experiments suggest a time window of S-phase to G2 for CENP-O loading to kinetochores. Also for CENP-T and -W [61], CENP-N [44] and CENP-U [62] loading to kinetochores in S-phase was observed.

**Figure 6:**
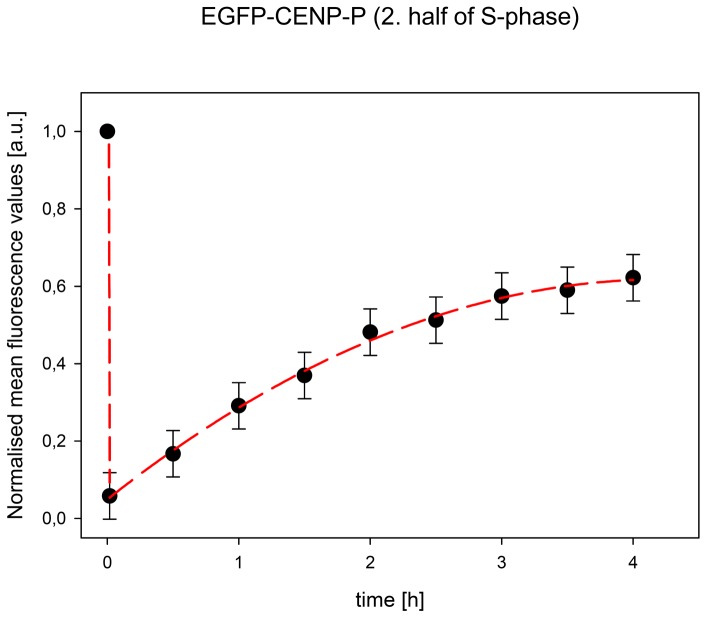
Fluorescence recovery after photobleaching of EGFP-CENP-P in mid S-phase. Normalised mean fluorescence values of 55 kinetochores taken in time steps of 30 min over 4 hours. Recovery levels off, indicative of an about 40% immobile fraction.

Our recent work showed that CENP-T and -W [61] as well as CENP-N [44] are loaded to human kinetochores by slow loading dynamics, mainly during the second half of S-phase. This is in contrast to CENP-A which is loaded at the end of mitosis and G1 [55], [60]. We speculated that the CENP-O class proteins might also be loaded slowly, mainly in S-phase. We thus studied the dynamic binding of these EGFP-tagged CENPs by Fluorescence Recovery After Photobleaching (FRAP) in living human U2OS cells. For none of the five CENP-O class proteins, at any cell cycle phase, we could detect fluorescence recovery within 150 sec after bleaching, indicating rather stable kinetochore binding of all five proteins, consistent with observations of Minoshima et al. [38] for CENP-U. We then studied fluorescence recovery of these five proteins during the cell cycle in a longer time frame, now over 4 hours. Different cell cycle phases were identified by staining with CENP-F and by co-expressing mRFP-PCNA for identifying S-phase and its sub-phases [63], [64], as recently described [44], [55], [65]. In G1, all five CENP-O class proteins show complete recovery; four proteins have an exchange rate (t_1/2_) of about one hour while only CENP-R exchanges slower with t_1/2_ = 2 hrs. In S-phase and G2, CENP-O, -P and -Q show partial recovery values of 40 to 80% with a slower exchange rate compared to G1 of about 2 hrs (see Table 3 and Fig. 6; in same cases for CENP-O and -P, the recovery only allows to estimate the final recovery level (values in brackets)). These recovery amplitudes are in the same range of values as for those of CENP-T and -W (70±8%) [61] and CENP-N (45±6%, see Table 3) [44]. The slow recovery times during the second half of S-phase coincide with the slow recovery times of CENP-T and -W (t_1/2_ = 70±10 min) [61], but are slower than the exchange of CENP-N (t_1/2_ = 38±7 min) [44]. In G2, CENP-P and -Q seem to show slightly faster recovery times compared to S-phase. The FRAP dynamics of CENP-U and -R are distinct from that of CENP-O, -Q and -P. CENP-U shows 100% recovery throughout the cell cycle with the exception of late S-phase when most of CENP-U (71±2%) is immobile (the remaining 29% of CENP-U exchange with t_1/2_ = 50±8 min). Our FRET data indicate that CENP-U di- or multimerises in late S-phase. This CENP-U self-assembly could reduce CENP-U exchange at the kinetochore in late S-phase, explaining the high immobile fraction detected by FRAP. This CENP-U/-U interaction seems not be mediated by Plk1 since Plk1 binding to kinetochores occurs during late G2 [41]. Our data indicate that CENP-Q and CENP-U form di- or oligomers after kinetochore binding before the onset of mitosis, potentially denoting a conformational change.

**Table 3:** Long term FRAP results for the CENP-PORQU proteins.

Cell cycle	CENP-O		CENP-P		CENP-Q		CENP-U		CENP-R	
	rec/%	t_1/2_/min	rec/%	t_1/2_/min	rec/%	t_1/2_/min	rec/%	t_1/2_/min	rec/%	t_1/2_/min
G1	100	71±15	100	77±15	90±10	57±10	100	72±15	100	125±15
early S	(45)	-	(70)	-	59±6	118±15	100	163±40	100	147±20
mid S	(40)	-	62±6	81±5	65±6	125±30	100	93±15	100	160±15
late S	75±15	131±30	49±10	103±10	75±8	136±15	29±2	50±8	100	180±20
G2	(80)	-	56±8	78±5	64±6	90±14	100	76±8	100	137±20

rec: fluorescence recovery relative to the initial fluorescence value before bleaching, t_1/2_: time for half height recovery (in min). Recovery values in brackets: estimated recovery value; for these data, a t_1/2_ value could not be determined.

Different from the behaviour of the other four proteins, for all cell cycle phases CENP-R shows recovery values of 100% with slow loading times of 2 to 3 hrs (Table 3). Thus, CENP-R recovery is considerably slower than that of the other four CENP-O class proteins. The observed distinct dynamical behaviour of the CENP-O class proteins indicates that the complex does not bind to the kinetochore as a pre-formed complex in the nucleoplasm and that these proteins retain distinct dynamic behaviour also when bound to the kinetochore.

### Cell-cycle dependent protein abundance

**Figure 7:**
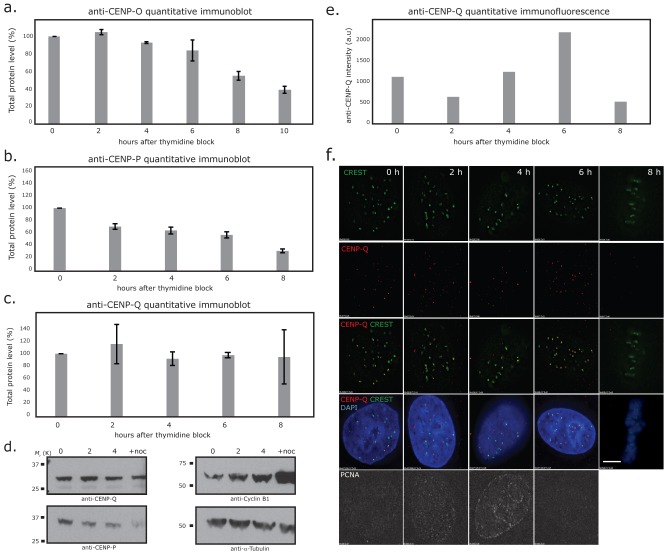
Levels of CENP-O/P/Q total protein during the cell cycle. (A) Quantitative immunoblot of CENP-O relative to α-Tubulin. Protein amounts are measured at G1/S (0 h), 2, 4, 6, 8 and 10 hrs after release from the double thymidine block in synchronised human HEp-2 cells. CENP-F and PCNA staining identify the time points 2, 4, and 6 hrs as S-phase, time point 8 hrs as G2 and 10 hrs as M-phase. The cellular amount of CENP-O reduces in G2 and further in M-phase. (B, C) Quantitative immuno-blots of CENP-P and CENP-Q protein levels relative to α-Tubulin at 0 (G1/S), 2 (early S), 4 (middle S), 6 (late S-phase), 8 (G2) hrs after release from double thymidine block in synchronized HeLa cells. Cycle stages were attributed from FACs analysis, PCNA staining and phase contrast microscopy (data not shown). (D) Representative immunoblots showing CENP-P, CENP-Q, Cyclin-B1 and α-Tubulin at the 0 (G1/S), 2 (early S), 4 (middle S) hrs time points and cells arrested in mitosis with nocodazole (16 hrs). (E) Quantitative four-colour immuno-flourence using anti-CENP-Q (red), CREST (green), DAPI (blue) and anti-PCNA (far red) antibodies in the same cells used in panel B. Pixel intensities of CENP-Q (signal – background) at kinetochores (n = 50 from 5 cells) are shown for each time point after release from double thymidine block (E) and representative images (F). CENP-Q loads onto kinetochores during S-phase reaching maximal binding in late S-phase (6 h). Scale bar = 5 µm.

The CCAN protein CENP-N shows varying abundance in the cell with a maximal protein level at kinetochores in late S-phase [8], [44]. Furthermore, the presence of CENP-U at HeLa kinetochores increases during late G1 and early S-phase, remains high through late S and G2 and decreases strongly during M-phase [40]. For human CENP-O, a decrease in kinetochore presence down to about 60% from interphase to mitosis was detected by immuno-fluorescence [33]. Here we extended these CENP-O data and measured the cell cycle dependent amount of CENP-O relative to tubulin in HEp-2 cells by Western blot 2, 4, 6 (S-phase), 8 (G2), and 10 hours (M-phase) after release from a double thymidine block (Fig. 7A). The cellular amount of CENP-O remains rather stable from G1/S over the entire S-phase, is reduced already in G2 and reduces further in M-phase, consistent with findings of McAinsh et al. [33]. A corresponding Western blot analysis was conducted for CENP-P and CENP-Q: The level of CENP-P decreases from late S-phase through G2 to M-phase (Fig. 7B, D), whereas CENP-Q displayed stable protein levels from G1/S into mitosis (Fig. 7C, D). In contrast to the constant level of CENP-Q levels in the cell, immune-fluorescence detected an increase of the amounts of CENP-Q at kinetochores during S-phase, reaching a maximum in late S-phase and strongly decreasing in G2 (Fig. 7E, F).

## Discussion

The centromeric histone H3 variant CENP-A is the central marker of centromere location and inherits this location to daughter cells [55]. The kinetochore recognizes this epigenetic mark, in part, through the CCAN network of proteins. The CENP-N subunit directly binds the CENP-A CATD region of the CENP-A containing nucleosome while the CENP-C subunit binds the C-terminal tail of CENP-A [14]–[16]. In addition to these CENP-A binding mechanisms, CENP-T/W/S/X form a unique centromeric chromatin structure next to histone H3 containing nucleosomes that supercoils DNA [9], [12], [61]. If we are to fully understand the pathways and mechanisms that allow a mature kinetochore to assemble, it will be crucial to define how these chromatin-interacting complexes recruit the other 11 CCAN subunits. Of these subunits the CENP-PORQU were reported to form a stable complex when being expressed in *E. coli* [10], whereas the CENP-H, -I, -K, -L and -M (CENP-H class) are not known to associate into any stable sub-complexes [8]. Dependency experiments show that CENP-PORQU requires the CENP-H class for kinetochore binding but not *vice versa* [5], [6], [8], [14], [52]. The working model thus involves the stepwise recruitment of CENP-N/CENP-TWSX≫CENP-HIKLM≫CENP-PORQU [2]. In line with this, CENP-L can bind directly to CENP-N *in vitro* [14] and may be involved in stabilising CENP-N binding to the CENP-A nucleosome [44]. We now show by F3H that CENP-N, CENP-K and CENP-L are all, to some extent, capable of recruiting CENP-O, -U and -R to an ectopic chromosomal site, whereas CENP-C is not directly involved in CENP-PORQU binding. The next step will be to identify the physical interactions that mediate this assembly reaction.

The CENP-PORQU proteins assemble at kinetochores during S-phase. For example, newly synthesized CENP-O is incorporated in S-phase and remains at the kinetochore during mitosis (although levels decrease, consistent with previous findings [33]) into the following G1 where they can exchange slowly and to near completion (however, without exchange with newly synthesized CENP-O). During the cell cycle, the CENP-PORQU proteins show different protein abundance in the cell: while CENP-Q protein levels do not change from G1/S to M-phase, the levels of CENP-O and -P decrease, CENP-P levels already during S-phase but those of CENP-O only after S-phase. The protein amount at kinetochores is maximal in late S-phase for CENP-Q, as shown here, and at late S-phase and G2 for CENP-U [40]. This variance of protein abundance in the cell and at kinetochores supports our conclusion that the CENP-PORQU complex assembles from proteins with individual behavior, and might indicate a varying stoichiometry of the CENP-PORQU proteins in the complex.

The reported stable interaction of CENP-PORQU in *E. coil* lysates [10] suggests that these proteins may form a pre-assembled complex in the nucleoplasm before loading onto kinetochores in S-phase. We show here, however, by FCCS that the CENP-PORQU subunits do not exist as a single preformed complex prior to kinetochore-binding. Instead, in the nucleoplasm, we can only detect a CENP-O/P (to an amount of about 50%), and, to a very minor extent, a CENP-Q/R heterodimer. However, by F3H we could show that each CENP-PORQU subunit can recruit two or three other proteins of this group to an ectopic chromosomal site. This confirms that these proteins specifically interact with each other in mammalian cells. One caveat of this experiment is that CCAN proteins might be specifically modified at centromere locations. These centromere specific modifications would be absent at the ectopic chromosomal site, potentially influencing protein interactions. Since pair-wise binding is weak in most cases, the strong kinetochore binding of the CENP-PORQU subunits (identified by slow FRAP recovery times) supports multi-fold CENP-PORQU interactions at the kinetochore. No single subunit of CENP-PORQU can recruit all other subunits, further supporting our finding that the complex does not pre-form in the nucleoplasm. Our FRAP experiments, consistent with previous studies [38], show that the cell cycle dependent turnover of CENP-P/O/Q is similar but distinct from the behavior of CENP-U and CENP-R. This indicates that the CENP-PORQU sub-complex does not behave as a single unit but instead is an ensemble of autonomously behaving proteins.

Our FRET measurements show that the CENP-PORQU proteins, once bound and incorporated into the inner kinetochore structure, are positioned in close proximity to one another. Previously, we reported that the amino-terminus of CENP-U was in close proximity to the amino-terminus of CENP-B and CENP-I, but not to the amino-terminus of CENP-A and CENP-C [43]. This indicates that, to some extent, CENP-PORQU is imbedded within the CCAN complex. Moreover, not all FRET connectivities should be thought of as occurring necessarily within a single CCAN inner kinetochore complex (intra-CCAN FRET). It is possible that some observed FRET proximities may reflect protein neighborhoods between two different adjacent CCAN complexes (inter-CCAN FRET). Such inter-CCAN interactions are likely, given super-resolution experiments that support models in which kinetochores are formed from multiple adjacent microtubule binding sites [66]–[68]. We expect that three-dimensional inner kinetochore model building will allow us to evaluate and explore these ideas.

The multifold interactions of the CENP-PORQU proteins result in stable binding of these proteins to kinetochores, suggesting a self-assembly mechanism [69]. In this regard, CENP-U and CENP-R are able to homo-dimerise at an ectopic chromosomal site (see Table 2), although we could not detect homo-dimerisation from our FCCS measurements. Nevertheless, upon kinetochore binding and before mitosis, these proteins are proximal to themselves, as detected by FRET between CENP-U/-U. Human CENP-Q, when expressed in *E. coli*, oligomerises into octameric complexes [36]. In late S-phase, after kinetochore binding and before mitosis, we detected FRET between the CENP-Q carboxy- as well as amino-terminal regions, indicating homo-di- or oligomerisation. We could not detect such homo-dimerisation at an ectopic chromosomal site, and found by FCCS that CENP-Q migrates as a monomer in the nucleoplasm, showing that the oligomerization event occurs at kinetochores. This self-association of CENP-U and -Q might hint towards the presence of more than one of these proteins (CENP-U, -Q) in one CCAN complex, indicating a varying stoichiometry in the complex. Alternatively, these proteins might make inter-CCAN interactions with themselves. Such an interaction between different CCAN complexes might induce or stabilize centromere specific chromatin structures and/or microtubule binding sites [70]. The latter hypothesis is attractive given that both CENP-Q and CENP-U bind directly to microtubules *in vitro* [36], [39]. We speculate that this self-association of CENP-Q and -U after kinetochore binding is a pre-mitotic maturation process that might switch kinetochores into the correct conformation for microtubule attachment.

## References

[1] Perpelescu M, Fukagawa T (2011) The ABCs of CENPs. Chromosoma 120: 425–446. 21751032 10.1007/s00412-011-0330-0

[2] Takeuchi K, Fukagawa T (2012) Molecular architecture of vertebrate kinetochores. Exp Cell Res 318: 1367–1374. 22391098 10.1016/j.yexcr.2012.02.016

[3] Cheeseman IM, Desai A (2008) Molecular architecture of the kinetochore-microtubule interface. Nat Rev Mol Cell Biol 9: 33–46. 18097444 10.1038/nrm2310

[4] Przewloka M, Glover DM (2009) The kinetochore and the centromere: a working long distance relationship. Annu Rev Genet 43: 439–465. 19886809 10.1146/annurev-genet-102108-134310

[5] Foltz DR, Jansen LET, Black BE, Bailey AO, YatesIII JR, et al (2006) The human CENP-A centromeric complex. Nat Cell Biol 8: 458–469. 16622419 10.1038/ncb1397

[6] Okada M, Cheeseman IM, Hori T, Okawa K, McLeod IX, et al (2006) The CENP-H-I complex is required for the efficient incorporation of newly synthesized CENP-A into centromeres. Nature Cell Biol 8: 446–457. 16622420 10.1038/ncb1396

[7] Meraldi P, McAinsh AD, Rheinbay E, Sorger PK (2006) Phylogenetic and structural analysis of centromeric DNA and kinetochore proteins. Genome Biol 7: R23. 16563186 10.1186/gb-2006-7-3-r23 PMC1557759

[8] McClelland SE, Borusu S, Amaro AC, Winter JR, Belwal M, et al (2007) The CENP-A NAC/CAD kinetochore complex controls chromosome congression and spindle bipolarity. EMBO J 26: 5033–5047. 18007590 10.1038/sj.emboj.7601927 PMC2140114

[9] Hori T, Amano M, Suzuki A, Backer CB, Welburn JP, et al (2008) CCAN makes multiple contacts with centromeric DNA to provide distinct pathways to the outer kinetochore. Cell 135: 1039–1052. 19070575 10.1016/j.cell.2008.10.019

[10] Hori T, Okada M, Maenaka K, Fukagawa T (2008) CENP-O class proteins form a stable complex and are required for proper kinetochore function. Mol Biol Cell 19: 843–854. 18094054 10.1091/mbc.E07-06-0556 PMC2262965

[11] Amano M, Suzuki A, Hori T, Backer C, Okawa K, et al (2009) The CENP-S complex is essential for the stable assembly of outer kinetochore structure. J Cell Biol 186: 173–182. 19620631 10.1083/jcb.200903100 PMC2717651

[12] Nishino T, Takeuchi K, Gascoigne KE, Suzuki A, Hori T, et al (2012) CENP-T-W-S-X forms a unique centromeric chromatin structure with a histone-like fold. Cell 148: 487–501. 22304917 10.1016/j.cell.2011.11.061 PMC3277711

[13] Santaguida S, Musacchio A (2009) The life and miracles of kinetochores. EMBO J 28: 2511–2531. 19629042 10.1038/emboj.2009.173 PMC2722247

[14] Carroll CW, Silva MCC, Godek KM, Jansen LET, Straight AF (2009) Centromere assembly requires the direct recognition of CENP-A nucleosomes by CENP-N. Nat Cell Biol 11: 896–902. 19543270 10.1038/ncb1899 PMC2704923

[15] Carroll CW, Milks KJ, Straight AF (2010) Dual recognition of CENP-A nucleosomes is required for centromere assembly. J Cell Biol 189: 1143–1155. 20566683 10.1083/jcb.201001013 PMC2894454

[16] Guse A, Carroll CW, Moree B, Fuller CJ, Straight AF (2011) In vitro centromere and kinetochore assembly on defined chromatin templates. Nature 477: 354–358. 21874020 10.1038/nature10379 PMC3175311

[17] Tachiwana H, Kagawa W, Shiga T, Osakabe A, Miya Y, et al (2011) Crystal structure of the human centromeric nucleosome containing CENP-A. Nature 476: 232–235. 21743476 10.1038/nature10258

[18] Bui M, Dimitriadis EK, Hoischen C, An E, Quenet D, et al (2012) Cell cycle-dependent structural transitions in the human CENP-A nucleosome in vivo. Cell 150: 317–326. 22817894 10.1016/j.cell.2012.05.035 PMC3592566

[19] De Wulf P, McAinsh AD, Sorger PK (2003) Hierarchical assembly of the budding yeast kinetochore from multiple subcomplexes. Genes Dev 17: 2902–2921. 14633972 10.1101/gad.1144403 PMC289150

[20] Cheeseman IM, Niessen S, Anderson S, Hyndman F, Yates JR 3rd, et al (2004) A conserved protein network controls assembly of the outer kinetochore and its ability to sustain tension. Genes Dev 18: 2255–2268. 15371340 10.1101/gad.1234104 PMC517519

[21] Cheeseman IM, Chappie JS, Wilson-Kubalek EM, Desai A (2006) The conserved KMN network constitutes the core microtubule-binding site of the kinetochore. Cell 127: 983–997. 17129783 10.1016/j.cell.2006.09.039

[22] Obuse C, Yang H, Nozaki N, Goto S, Okazaki T, et al (2004) Proteomics analysis of the centromere complex from HeLa interphase cells: UV-damaged DNA binding protein 1 (DDB-1) is a component of the CEN-complex, while BMI-1 is transiently co-localised with the centromeric region in interphase. Genes to Cells 9: 105–120. 15009096 10.1111/j.1365-2443.2004.00705.x

[23] Liu X, McLeod I, Anderson S, Yates JR 3rd, He X (2005) Molecular analysis of kinetochore architecture in fission yeast. EMBO J 24: 2919–2930. 16079914 10.1038/sj.emboj.7600762 PMC1187945

[24] Petrovic A, Pasqualato S, Dube P, Krenn V, Santaguida S, et al (2010) The Mis12 complex is a protein interaction hub for outer kinetochore assembly. J Cell Biol 190: 835–852. 20819937 10.1083/jcb.201002070 PMC2935574

[25] Kiyomitsu T, Iwasaki O, Obuse C, Yanagida M (2010) Inner centromere formation requires hMis14, a trident kinetochore protein that specifically recruits HP1 to human chromosomes. J Cell Biol 188: 791–807. 20231385 10.1083/jcb.200908096 PMC2845078

[26] Liu D, Vleugel M, Backer CB, Hori T, Fukagawa T, et al (2010) Regulated targeting of protein phosphatise 1 to the outer kinetochore by KNL1 opposes Aurora B kinase. J Cell Biol 188: 809–820. 20231380 10.1083/jcb.201001006 PMC2845083

[27] Przewloka MR, Venkei Z, Bolanos-Garcia VM, Debski J, et al (2011) CENP-C is a structural platform for kinetochore assembly. Curr Biol 21: 399–405. 21353555 10.1016/j.cub.2011.02.005

[28] Tanaka TU, Desai A (2008) Kinetochore-microtubule interactions: the means to the end. Curr Opin Cell Biol 20: 53–63. 18182282 10.1016/j.ceb.2007.11.005 PMC2358929

[29] Wan X, O'Quinn RP, Pierce HL, Joglekar AP, Gall WE, et al (2009) Protein architecture of the human kinetochore microtubule attachment site. Cell 137: 672–684. 19450515 10.1016/j.cell.2009.03.035 PMC2699050

[30] Moree B, Meyer CB, Fuller CJ, Straight AF (2011) CENP-C recruits M18BP1 to centromeres to promote CENP-A chromatin assembly. J Cell Biol 194: 855–871. 21911481 10.1083/jcb.201106079 PMC3207292

[31] Barnhart MC, Kuich HJL, Stellfox ME, Ward JA, Bassett EA, et al (2011) HJURP is a CENP-A chromatin assembly factor sufficient to form a functional de novo kinetochore. J Cell Biol 194: 229–243. 21768289 10.1083/jcb.201012017 PMC3144403

[32] Fukagawa T, Mikami Y, Nishihashi A, Regnier V, Haraguchi T, et al (2001) CENP-H, a constitutive centromere component, is required for centromere targeting of CENP-C in vertebrate cells. EMBO J 20: 4603–4617. 11500386 10.1093/emboj/20.16.4603 PMC125570

[33] McAinsh AD, Meraldi P, Draviam VM, Toso A, Sorger PK (2006) The human kinetochore proteins Nnf1R and Mcm21R are required for accurate chromosome segregation. EMBO J 25: 4033–4049. 16932742 10.1038/sj.emboj.7601293 PMC1560365

[34] Toso A, Winter JR, Garrod AJ, Amaro AC, Meraldi P, et al (2009) Kinetochore-generated pushing forces separate centrosomes during bipolar spindle assembly. J Cell Biol 184: 365–372. 19204145 10.1083/jcb.200809055 PMC2646558

[35] Gascoigne KE, Takeuchi K, Suzuki A, Hori T, Fukagawa T, et al (2011) Induced ectopic kinetochore assembly bypasses the requirement for CENP-A nucleosomes. Cell 145: 410–422. 21529714 10.1016/j.cell.2011.03.031 PMC3085131

[36] Amaro AC, Samora CP, Holtackers R, Wang E, Kingston IJ, et al (2010) Molecular control of kinetochore-microtubule dynamics and chromosome oscillations. Nature Cell Biol 12: 319–329. 20228811 10.1038/ncb2033 PMC2909587

[37] Izuta H, Ikeno M, Suzuki N, Tomonaga T, Nozaki N, et al (2006) Comprehensive analysis of the ICEN (Interphase Centromere Complex) components enriched in the CENP-A chromatin of human cells. Genes Cells 11: 673–684. 16716197 10.1111/j.1365-2443.2006.00969.x

[38] Minoshima Y, Hori T, Okada M, Kimura H, Haraguchi T, et al (2005) The constitutive centromere component CENP-50 is required for recovery from spindle damage. Mol Cell Biol 25: 10315–10328. 16287847 10.1128/MCB.25.23.10315-10328.2005 PMC1291240

[39] Hua S, Wang Z, Jiang K, Huang Y, Ward T, et al (2011) CENP-U cooperates with Hec1 to orchestrate kinetochore-microtubule attachment. J Biol Chem 286: 1627–1638. 21056971 10.1074/jbc.M110.174946 PMC3020771

[40] Kang YH, Park JE, Yu LR, Soung NK, Yun SM, et al (2006) Self-regulated Plk1 recruitment to kinetochores by the Plk1-PBIP1 interaction is critical for proper chromosome segregation. Mol Cell 24: 409–422. 17081991 10.1016/j.molcel.2006.10.016

[41] Kang YH, Park C-H, Kim T-S, Soung N-K, Bang JK, et al (2011) Mammalian Polo-like kinase 1-dependent regulation of the PBIP1-CENP-Q complex at kinetochores. J Biol Chem 286: 19744–19757. 21454580 10.1074/jbc.M111.224105 PMC3103353

[42] Schmitzberger F, Harrison SC (2012) RWD domain: a recurring module in kinetochore architecture shown by Ctf19-Mcm21 complex structure. EMBO Rep 13: 216–222. 22322944 10.1038/embor.2012.1 PMC3323139

[43] Hellwig D, Hoischen C, Ulbricht T, Diekmann S (2009) Acceptor-photobleaching FRET analysis of core kinetochore and NAC proteins in living human cells. Eur Biophys J 38: 781–791. 19533115 10.1007/s00249-009-0498-x

[44] Hellwig D, Emmerth S, Ulbricht T, Doering V, Hoischen C, et al (2011) Dynamics of CENP-N kinetochore binding during the cell cycle. J Cell Sci 124: 3871–3883. 22100916 10.1242/jcs.088625 PMC3225271

[45] Tsukamoto T, Hashiguchi N, Janicki SM, Tumbar T, Belmont AS, et al (2000) Visualization of gene activity in living cells. Nat Cell Biol 2: 871–878. 11146650 10.1038/35046510

[46] Rothbauer U, Zolghadr K, Tillib S, Nowak D, Schermelleh L, et al (2006) Targeting and tracing antigens in live cells with fluorescent nanobodies. Nat Methods 3: 887–889. 17060912 10.1038/nmeth953

[47] Zolghadr K, Mortusewicz O, Rothbauer U, Kleinhans R, Goehler H, et al (2008) A fluorescent two-hybrid assay for direct visualization of protein interactions in living cells. Mol Cell Proteomics 7: 2279–2287. 18622019 10.1074/mcp.M700548-MCP200

[48] Orthaus S, Biskup C, Hoffmann B, Hoischen C, Ohndorf S, et al (2008) Assembly of the inner kinetochore proteins CENP-A and CENP-B in living human cells. Chem Bio Chem 9: 77–92. 10.1002/cbic.200700358 18072184

[49] Bacia K, Schwille P (2003) A dynamic view of cellular processes by *in vivo* fluorescence auto- and cross-correlation spectroscopy. Methods 29: 74–85. 12543073 10.1016/s1046-2023(02)00291-8

[50] Bacia K, Schwille P (2007) Practical guidelines for dual-color fluorescence cross-correlation spectroscopy. Nature Protocols 2.28422856. 10.1038/nprot.2007.410 18007619

[51] Orthaus S, Klement K, Happel N, Hoischen C, Diekmann S (2009) Linker Histone H1 is present in centromeric chromatin of living human cells next to inner kinetochore proteins. Nucl Acids Res 37: 3391–3406. 19336418 10.1093/nar/gkp199 PMC2691837

[52] Milks KJ, Moree B, Straight AF (2009) Dissection of CENP-C directed centromere and kinetochore assembly. Mol Biol Cell 20: 4246–4255. 19641019 10.1091/mbc.E09-05-0378 PMC2754938

[53] Qui SL, Wang JN, Yu C, He DC (2009) CENP-K and CENP-H may form coiled-coils in the kinetochores. Sci China Ser C-Life Sci 52: 352–359. 19381461 10.1007/s11427-009-0050-3

[54] Digman MA, Brown CM, Sengupta P, Wiseman PW, Horwitz AR, et al (2005) Measuring fast dynamics in solutions and cells with a laser scanning microscope. Biophys J 89: 1317–1327. 15908582 10.1529/biophysj.105.062836 PMC1366616

[55] Hemmerich P, Weidtkamp-Peters S, Hoischen C, Schmiedeberg L, Erliandri I, et al (2008) Dynamics of inner kinetochore assembly and maintenance in living cells. J Cell Biol 180: 1101–1114. 18347072 10.1083/jcb.200710052 PMC2290840

[56] Kohl T, Haustein E, Schwille P (2005) Determining protease activity *in vivo* by fluorescence cross-correlation analysis. Biophys J 89: 2770–2782. 16055538 10.1529/biophysj.105.061127 PMC1366777

[57] Hanissian SH, Akbar U, Teng B, Janjetovic Z, Hoffmann A, et al (2004) cDNA cloning and characterization of a novel gene encoding the MLF1-interacting protein MLF1IP. Oncogene 23: 3700–3707. 15116101 10.1038/sj.onc.1207448

[58] Suzuki H, Arakawa Y, Ito M, Saito S, Takeda N, et al (2007) MLF1-interacting protein is mainly localized in nucleolus through N-terminal bipartite nuclear localization signal. Anticancer Res 27: 1423–1430. 17595757

[59] Keppler A, Gendreizig S, Gronemeyer T, Pick H, Vogel H, et al (2003) A general method for the covalent labelling of fusion proteins with small molecules *in vivo* . Nat Biotechnol 21: 86–89. 12469133 10.1038/nbt765

[60] Jansen LET, Black BE, Foltz DR, Cleveland DW (2007) Propagation of centromeric chromatin requires exit from mitosis. J Cell Biol 176: 795–805. 17339380 10.1083/jcb.200701066 PMC2064054

[61] Prendergast L, van Vuuren C, Kaczmarczyk A, Döring V, Hellwig D, et al (2011) Premitotic assembly of human CENPs -T and -W switches centromeric chromatin to a mitotic state. PloS Biol 9: e1001082. 21695110 10.1371/journal.pbio.1001082 PMC3114758

[62] Lee KS, Oh DY, Kang YH, Park JE (2008) Self-regulated mechanism of Plk1 localisation to kinetochores: lessons from the Plk1-PBIP1 interaction. Cell Div 3: 4. 18215321 10.1186/1747-1028-3-4 PMC2263035

[63] Leonhardt H, Rahn HP, Weinzierl P, Sporbert A, Cremer T, et al (2000) Dynamics of DNA replication factories in living cells. J Cell Biol 149: 271–280. 10769021 10.1083/jcb.149.2.271 PMC2175147

[64] Sporbert A, Domaing P, Leonhardt H, Cardoso MC (2005) PCNA acts as a stationary loading platform for transiently interacting Okazaki fragment maturation proteins. Nucleic Acids Res 33: 3521–3528. 15972794 10.1093/nar/gki665 PMC1156965

[65] Schmiedeberg L, Weisshart K, Diekmann S, Meyer zu Hoerste G, Hemmerich P (2004) High- and low-mobility populations of HP1 in heterochromatin of mammalian cells. Mol Biol Cell 15: 2819–2833. 15064352 10.1091/mbc.E03-11-0827 PMC420105

[66] Ribeiro SA, Vagnarelli P, Dong Y, Hori T, McEwen BF, et al (2010) A super-resolution map of the vertebrate kinetochore. Proc Natl Acad Sci USA 107: 10484–10489. 20483991 10.1073/pnas.1002325107 PMC2890832

[67] Johnston K, Joglekar A, Hori T, Suzuki A, Fukagawa T, Salmon ED (2010) Vertebrate kinetochore protein architecture: protein copy number. J Cell Biol 189: 937–943. 20548100 10.1083/jcb.200912022 PMC2886349

[68] Lawrimore J, Bloom KS, Salmon ED (2011) Point centromeres contain more than a single centromere-specific Cse4 (CENP-A) nucleosome. J Cell Biol 195: 573–582. 22084307 10.1083/jcb.201106036 PMC3257525

[69] Hemmerich P, Schmiedeberg L, Diekmann S (2011) Dynamic as well as stable protein interactions contribute to genome function and maintenance. Chromosome Res 19: 131–151. 21046224 10.1007/s10577-010-9161-8 PMC3040344

[70] Dong Y, VandenBeldt KJ, Meng X, Khodjakov A, McEwen BF (2010) The outer plate in vertebrate kinetochores is a flexible network with multiple microtubule interactions. Nat Cell Biol 9: 516–522. 10.1038/ncb1576 PMC2895818 17435749

